# Complications in Thoracic Minimally Invasive Spine Surgery (2013–2024): A Systematic Review

**DOI:** 10.3390/jcm15010363

**Published:** 2026-01-03

**Authors:** Sean Inzerillo, Chibuikem A. Ikwuegbuenyi, Eesha Gurav, Noah Willett, Mousa Hamad, Ibrahim Hussain, Alan Hernández-Hernández, Galal Elsayed, Osama Kashlan, Roger Härtl

**Affiliations:** 1 College of Medicine, State University of New York Downstate Health Sciences University, Brooklyn, NY 11203, USA; sean.inzerillo@downstate.edu; 2 Department of Neurological Surgery, Och Spine at New York-Presbyterian Hospital, Weill Cornell Medicine, New York, NY 10021, USA; cai4001@med.cornell.edu (C.A.I.); now4003@med.cornell.edu (N.W.); mkh4002@med.cornell.edu (M.H.); ibh9004@med.cornell.edu (I.H.); sbk9015@med.cornell.edu (G.E.); onk4001@med.cornell.edu (O.K.); 3 School of Medicine, University of South Carolina, Columbia, SC 29208, USA; eesha.gurav@uscmed.sc.edu; 4 Department of Neurosurgery, Instituto Nacional de Neurología y Neurocirugía Manuel Velasco Suárez, Mexico City 14269, Mexico; drahh9208@gmail.com

**Keywords:** thoracic spine, minimally invasive spine surgery, tubular retractor, uniportal endoscopic surgery, biportal endoscopic surgery, complications

## Abstract

**Background/Objectives**: Thoracic minimally invasive spine surgery (MISS) offers reduced tissue trauma and faster recovery compared with open approaches, but its adoption remains limited due to technical complexity and uncertainty regarding complication rates. This study aimed to synthesize the available evidence on overall and approach-specific complications of thoracic MISS using tubular, uniportal endoscopic, and biportal endoscopic techniques. **Methods**: Following PRISMA guidelines (PROSPERO CRD42024594316), PubMed, Medline, Embase, and Cochrane Library were searched from January 2013 to March 2024 for studies reporting complication rates after thoracic MISS in adults. Eligible studies included tubular, uniportal, or biportal approaches. Study quality was assessed using the Newcastle–Ottawa Scale. Complication data were extracted and summarized descriptively, with reported rates stratified by surgical approach. **Results**: Nine studies (234 patients) were included, all retrospective with moderate risk of bias. Across included studies, reported overall complication rates following thoracic MISS ranged from 0% to 42.9%. Stratified by surgical approach, reported complication rates ranged from 0% to 11.8% across six tubular studies, 9.7% to 20.0% across two uniportal endoscopic studies, and 42.9% in a single small biportal endoscopic study. Neural injuries and dural tears were the most frequently reported complications, with reported rates ranging from 0% to 14.3% and 0% to 3.6%, respectively. Symptomatic cerebrospinal fluid leaks and revision surgeries were less common, with reported rates ranging from 0% to 11.8% and 0% to 3.2%, respectively. **Conclusions**: Thoracic MISS demonstrates a low overall complication rate, particularly for tubular approaches. However, findings should be interpreted with caution given the small number of included studies, retrospective designs, and clinical heterogeneity. Larger comparative investigations are needed to better define safety profiles and support broader adoption of thoracic MISS techniques.

## 1. Introduction

The introduction of minimally invasive spine surgery (MISS) has transformed spinal care by reducing tissue trauma and expediting recovery [1]. Yet, its adoption for thoracic pathologies has lagged compared to its widespread use in the cervical and lumbar spine [1,2]. This has been attributed to the unique challenges posed by the complex anatomy of this region, including the narrow spinal canal, limited operative corridor, and proximity to vital structures such as the spinal cord, major vascular structures, and pulmonary cavities [2,3]. However, thoracic MISS has gained momentum in recent years, with increasing utilization for indications such as disk herniations, stenosis, and tumors [4,5,6,7]. These conditions are being addressed using tubular retractors, uniportal endoscopic spine surgery (UESS), and biportal endoscopic spine surgery (BESS), which have demonstrated encouraging outcomes in safety and efficacy [4,8,9].

Despite recent advances, the steep learning curve associated with thoracic MISS poses a significant risk of complications and continues to hinder adoption in clinical practice [10,11,12]. Among documented complications, neural injuries occur most frequently, followed by dural tears and symptomatic cerebrospinal fluid (CSF) leaks, with incidence rates varying across different minimally invasive approaches [8,9,13]. However, the existing literature is fragmented, with complication data reported across diverse pathologies, patient cohorts, and operative techniques, limiting meaningful comparison of safety profiles in thoracic MISS.

To address this knowledge gap, we conducted a systematic review of the literature evaluating reported complication rates associated with thoracic MISS using tubular, uniportal, and biportal approaches. By synthesizing data from the past decade, this review provides a consolidated descriptive overview of complication patterns to inform surgical decision-making, contextualize procedural risk, and support ongoing refinement and adoption of thoracic MISS techniques.

## 2. Materials and Methods

Literature Search

We registered our study protocol in PROSPERO (CRD42024594316) and conducted our review following Preferred Reporting Items for Systematic Reviews and Meta-Analyses (PRISMA) guidelines [14]. PubMed, Medline, Embase via OVID, and the Cochrane Library were queried to comprehensively search the literature over the last decade (January 2013 through March 2024), employing Medical Subject Headings and relevant keywords to capture a broad range of publications related to thoracic MISS and its associated complications. The search terms included the following: “Minimally invasive,” “MISS,” “Minimally Invasive Surgical Procedures,” “tubular,” “biportal,” “uniportal,” “spine,” “surgery,” “complications,” “lumbosacral region,” “cervical,” “thoracic,” “lumbar,” “postoperative complications,” and “intraoperative complications.” The broad search terms enabled a comprehensive assessment of complications across MISS techniques (biportal, tubular, uniportal) and spinal regions (cervical, thoracic, lumbar), ensuring standardized screening while clearly distinguishing thoracic MISS studies in this review. A manual search via Google Scholar was also done to collect any relevant articles not initially uncovered during the database search. The full search strings used for each database are detailed in Supplementary Text S1, and the completed PRISMA 2020 checklist is provided in Supplementary Table S1.

Inclusion and Exclusion Criteria

Eligibility criteria selected for studies focusing on minimally invasive thoracic spine surgery utilizing a tubular retractor, uniportal endoscopic, or biportal endoscopic approach in adult patient cohorts of at least 10 individuals. The minimum cohort size was selected to exclude single-case or very small series, which may yield unstable complication estimates and limit generalizability, and this threshold aligns with recent work evaluating complication rates in cervical MISS [15]. A key requirement for inclusion was the availability of detailed complication data, either through the reported frequency of specific complications or an explicit indication that none occurred. Studies were excluded if they were conference abstracts, reviews, meta-analyses, or lacked an English full-text. In situations where multiple publications analyzed overlapping patient populations, the study with the larger sample size was prioritized for inclusion.

Study Screening and Data Collection

Following the literature search, retrieved studies were transferred into Rayyan (Rayyan Systems Inc., Cambridge, MA, USA), a web-based collaborative systematic review platform designed to facilitate the study screening process. Duplicate records were removed before screening. Two reviewers (S.I. and E.G.) independently assessed the titles and abstracts of the remaining articles against the pre-established inclusion and exclusion criteria. For studies considered potentially eligible, full texts were obtained and reviewed following the same procedure. Data extraction was then conducted using a standardized form to record key information such as study design, patient demographics, primary pathology treated, complication rates, and bias risk. To ensure accuracy, two reviewers (S.I. and E.G.) independently completed the extraction process. Any discrepancies between the reviewers were resolved through discussion, and if consensus was not reached, a third reviewer (C.I.) adjudicated the final decision.

Quality Assessment

The quality of the included studies was assessed using the Newcastle-Ottawa Scale (NOS) a tool specifically developed for evaluating non-randomized studies, such as cohort and case–control designs [16]. A score of 6 or greater on the NOS was classified as indicative of good study quality, following the criteria used in previous meta-analyses of biportal endoscopic spine surgery complications [17]. Reviewers S.I. and E.G. evaluated the quality of each study independently, with a third reviewer (C.I.) consulted to resolve any discrepancies and maintain evaluation consistency.

Data Processing and Statistical Analysis

The primary outcome was the reported incidence of overall complications following thoracic minimally invasive spine surgery, with secondary outcomes including the incidence of specific complications such as neural injury, dural tear, symptomatic cerebrospinal fluid leak, and surgical revision. Complication data were extracted directly from each included study and summarized descriptively, with complication rates calculated as the number of patients experiencing a given complication divided by the total number of patients in each study cohort. Reported complication rates were additionally stratified by surgical approach, including tubular, uniportal endoscopic, and biportal endoscopic techniques, and only outcomes reported by at least five studies across all surgical approaches were considered for analysis. Given the substantial clinical and methodological heterogeneity across studies, including variability in surgical indications, patient populations, and study design, formal pooled meta-analysis was not performed. Instead, results are presented as ranges of reported complication rates to facilitate transparent comparison while avoiding overinterpretation of heterogeneous data. All data management and descriptive analyses were performed using R version 4.4.1 (R Project for Statistical Computing, Vienna, Austria).

## 3. Results

### 3.1. Study Characteristics and Quality Assessment

**Figure 1:**
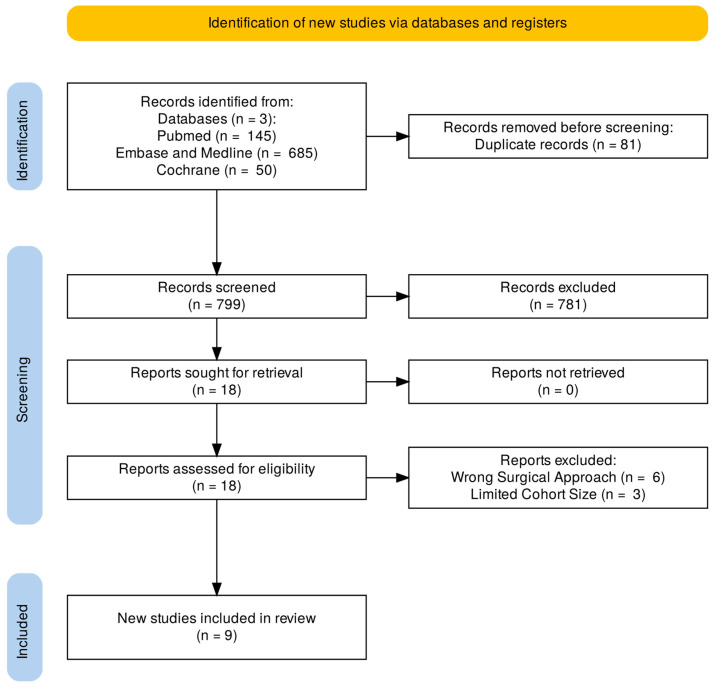
PRISMA Flow Diagram for study selection.

A total of 880 publications were returned in the initial database search, with 81 duplicates removed, leaving 799 studies for title and abstract screening. The PRISMA flow diagram for study selection is presented in Figure 1. Following this, 18 studies were selected for full-text review to identify the incidence of complications in thoracic MISS. Nine studies were eligible for inclusion, spanning 234 patients. The study characteristics and complication data are summarized in Table 1. The sample sizes ranged from 13 to 55 patients, and mean participant age ranged from 49.9 to 64.0 years. Males amounted to 40.3% of the participants in the seven studies that reported sex. Study follow-up periods ranged from 3 months to 29 months. Thoracic spinal tumors were examined in five studies, ossification of the ligamentum flavum in three, and disk herniation in two. Four of the nine included studies were conducted in China, making it the most represented country in this analysis. All the studies were retrospective, with all but one following a cohort design. Overall, the included studies were methodologically robust, with eight studies meeting the NOS thershold (≥6) indicative of good quality. A single cohort study was found to have a moderate risk of bias with a NOS score of 5/9. The quality assessment results are presented in Table 2.

### 3.2. Complication Rates

#### 3.2.1. Total Complications

**Figure 2:**
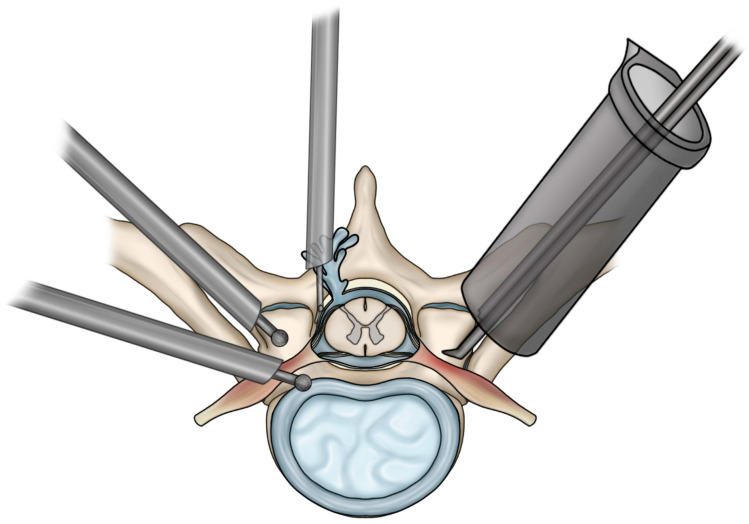
Illustration showing potential mechanisms of neural injury, dural tear, and cerebrospinal fluid leakage during tubular or endoscopic thoracic minimally invasive spine surgery.

Nine studies encompassing 234 patients reported overall complication outcomes following thoracic MISS, with 25 total complication events observed. Across all included studies, reported total complication rates ranged from 0% to 42.9%. Among tubular approaches, evaluated in six studies involving 134 patients, total complication rates ranged from 0% to 11.8%. The uniportal endoscopic approach, reported in two studies with 86 patients, demonstrated complication rates ranging from 9.7% to 20.0%. The biportal endoscopic approach, reported in a single study of 14 patients, demonstrated a complication rate of 42.9%. Figure 2 provides a schematic overview of intraoperative mechanisms that may underlie these complications, including dural tears, CSF leakage, and neural traction injury encountered during tubular and endoscopic thoracic MISS approaches.

#### 3.2.2. Neural Injuries

Seven studies encompassing 190 patients reported neural injury outcomes, with 10 total events observed. Reported neural injury rates across all thoracic MISS studies ranged from 0% to 14.3%. The tubular approach, reported in four studies involving 90 patients, demonstrated neural injury rates ranging from 0% to 10.0%. The uniportal endoscopic approach, reported in two studies including 86 patients, showed neural injury rates ranging from 0% to 10.9%. The biportal endoscopic approach, reported in a single study of 14 patients, demonstrated a neural injury rate of 14.3%.

#### 3.2.3. Dural Tears

Five studies encompassing 160 patients reported dural tear outcomes, with four total events observed. Across all thoracic MISS studies, reported dural tear rates ranged from 0% to 3.6%. The tubular approach, reported in two studies involving 60 patients, demonstrated dural tear rates ranging from 0% to 2.5%. The uniportal endoscopic approach, reported in two studies including 86 patients, showed dural tear rates ranging from 3.2% to 3.6%. No dural tears were reported in the single biportal endoscopic study involving 14 patients.

#### 3.2.4. Symptomatic CSF Leaks

Six studies encompassing 177 patients reported outcomes related to symptomatic CSF leaks, with three total events observed. For this study, symptomatic CSF leaks were defined as those causing external wound drainage, pseudomeningocele formation, or low-pressure headaches. The reported symptomatic CSF leak rate across all thoracic MISS studies ranged from 0% to 11.8%. The tubular approach, reported in four studies involving 108 patients, demonstrated symptomatic CSF leak rates ranging from 0% to 11.8%. The uniportal endoscopic approach, evaluated in one study of 55 patients, reported no symptomatic CSF leaks. The biportal endoscopic approach, reported in a single study of 14 patients, demonstrated a symptomatic CSF leak rate of 7.1%.

#### 3.2.5. Surgical Revisions

Five studies encompassing 154 patients reported outcomes related to revision surgery following thoracic MISS, with two total revision events observed. Across all included studies, reported revision rates ranged from 0% to 3.2%. The tubular approach, evaluated in three studies involving 68 patients, reported no revision surgeries. The uniportal endoscopic approach, reported in two studies with 86 patients, demonstrated revision rates ranging from 1.8% to 3.2%. Revision rate was not described in the single included study using a biportal approach.

## 4. Discussion

Our study provides a comprehensive synthesis of reported complication rates in thoracic minimally invasive spine surgery across nine studies encompassing 234 patients. This analysis offers valuable insights into the safety profile of thoracic MISS and facilitates comparisons with MISS applications in the cervical and lumbar regions. Across the included literature, neural injuries and dural tears were the most frequently reported complications.

Differences in reported complication rates across thoracic MISS approaches should be interpreted cautiously and may be influenced by the limited number of studies evaluating UESS (n = 2) and BESS (n = 1), which limits meaningful comparison with tubular techniques. In our review, BESS was used exclusively for ossification of the ligamentum flavum (OLF), while UESS was primarily used for thoracic disk herniations, one of the most technically challenging spinal pathologies [8,9]. Tubular approaches included a broader mix but were often used for stenosis and OLF [4,20]. Accordingly, the higher complication rate reported for BESS reflects a single small cohort treated for OLF and may not be representative when considered alongside lower rates reported for tubular and uniportal approaches treating similar pathology [9,19,20]. Thus, observed differences in complication rates may reflect underlying pathology rather than technique alone, likely confounding subgroup analyses.

By contrast, studies in lumbar spine surgery have directly compared minimally invasive subtypes such as UESS and BESS within single pathologies using standardized techniques. For example, in patients with lumbar spinal stenosis treated with unilateral laminectomy for bilateral decompression, UESS was associated with higher complication rates, although the difference was not statistically significant [21,22,23]. Similar technical challenges may exist in thoracic spine surgery, and prior studies have recommended building proficiency in full-endoscopic techniques at the lumbar level before advancing to thoracic procedures [8]. Furthermore, while comparative analyses of endoscopic and tubular retractor techniques are well-documented in lumbar spine surgery, such data remain unavailable for thoracic procedures [24,25]. The scarcity of both isolated and comparative complication data in thoracic MISS highlights the critical need for further research to support broader adoption and optimization of these advanced techniques.

Neural Injuries

Neural injuries were the most commonly reported complication in thoracic MISS across the included studies. While nerve injuries in the thoracic spine may be more tolerated due to segmental innervation patterns, they remain a concern, particularly at levels such as T1-2, where involvement of the sympathetic chain or brachial plexus contributions can lead to significant functional deficits [2,26]. Vascular compromise may also contribute to neurological injury risk in this region [27]. Thoracic MISS techniques add another layer of difficulty, given the narrow anatomical corridor and limited working space [26]. An earlier review of open thoracic spine surgery for ossification of the posterior ligament reported a postoperative neurological deficit rate of 13.9%, suggesting that thoracic MISS may offer advantages in reducing neurological complications, given the lower neural injury rate observed in our analysis [13]. Balasubramanian et al. observed a 10% neural injury rate in procedures employing tubular retractors, primarily involving tumors such as meningiomas, where dense adhesions to nerve roots complicated dissection and caused postoperative weakness, though recovery was achieved within six months [6]. Additionally, a large cohort study on UESS demonstrated similar neural injury rates in anterior transthoracic retropleural techniques compared to posterior extraforaminal and interlaminar approaches [8].

To minimize iatrogenic neural injury during thoracic MISS, precise manipulation of neural structures is essential. Preoperative adhesiolysis reduces tension on nerve roots, while limiting the use of high-intensity radiofrequency devices near neural elements helps mitigate the risk of thermal injury [9,28,29]. Particular attention should be given to the T1 nerve root to prevent inadvertent compression or damage, which may lead to hand weakness and disrupt sympathetic chain fibers, potentially resulting in Horner’s syndrome [30]. Proficiency in the chosen MISS technique, especially for endoscopic procedures, along with anatomical expertise and careful patient selection, is crucial for reducing neural injury complications [26].

Dural Tears

Dural tears are considered one of the most frequent complications in spine surgery, particularly in cases involving complex thoracic pathology [31]. A meta-analysis pooling data from six studies on complications following open laminectomy for thoracic myelopathy due to OLF reported a high dural tear rate of 18.4% [31]. More recently, Kim et al. compared outcomes of thoracic UESS laminotomy and decompression using the 1-block resection technique to open laminectomy for OLF, reporting a slightly lower dural tear rate of 3.2% in UESS patients compared to 5% in the open group [20]. These tears were managed intraoperatively using patch blocking with Tachosil, a technique that effectively sealed the dural breaches. The authors attributed the lower rate in UESS to improved endoscopic magnification and continuous irrigation, which enhanced visualization and precision during endoscopic drilling of OLF [20,31]. Similarly, Ruetten et al. reported a dural tear rate of 3.6% in thoracic UESS decompression, with two anterior dural leaks occurring during the resection of calcified disk herniations with adhesions. These leaks were managed using dural substitutes and fat flaps, as suturing was not feasible due to the limited access and the fragile condition of the dura in cases of calcified adhesions [8]. Favorable dural tear rates were also observed in thoracic tubular and BESS cases [4,9].

Symptomatic CSF Leaks

Although less common than dural tears, symptomatic CSF leaks are a recognized complication in thoracic spine surgery, often influenced by the degree of dural adhesion and ossification [32]. A large retrospective review of 266 cases of open thoracic OLF treatment reported a symptomatic CSF leak rate of 32%, frequently associated with complications such as pseudocysts, wound dehiscence, and meningitis [32]. However, a recent meta-analysis found a lower rate of 12.1% in open OLF surgery [31]. In our study, a single symptomatic CSF leak (7.1%) occurred in thoracic BESS for OLF, managed successfully with drainage removal, prone positioning, and pressure dressing, without further complications [9]. While no symptomatic CSF leaks were observed in the included UESS studies, two leaks (11.8%) were reported in tubular MISS for thoracic meningioma resection, reflecting the technical challenges of operating near adherent dura [8,18,20]. Overall, thoracic MISS appears to reduce delayed complications of symptomatic CSF leakage compared to open alternatives, as the absence of an extensive wound cavity to accumulate a pseudomeningocele and the limited routes for symptomatic CSF egress mitigate these risks [4].

Surgical Revisions

Surgical revisions were infrequently reported following thoracic MISS across the included studies. In open posterior spinal surgery for thoracic spinal stenosis, Hu et al. reported a 30-day unplanned revision rate of 3.95%, primarily attributed to epidural hematoma, wound-related complications, inadequate decompression, and implant malposition or failure [33]. Surgical factors, including upper thoracic spine involvement, thoracic kyphosis ≥ 45°, and intraoperative dural injury, were associated with higher revision risk. Thoracic MISS, with its enhanced direct visualization and restricted operative field that avoids unnecessary dissection of adjacent muscle and bone, may reduce the risk of postoperative complications, such as hematoma formation, by limiting the space for fluid accumulation and thereby decreasing the need for reoperation [9]. In our study, only two revision events were observed, both in UESS cases. One case required hematoma removal at T1–2, which resulted in complete pain relief. The other involved additional decompression of the superior articular process to achieve sufficient spinal canal widening, leading to successful symptom resolution [8,20]. Although revision rates in thoracic MISS were low, variability in revision surgery definitions, patient characteristics, and procedural complexity across studies should be carefully considered when interpreting these outcomes.

Limitations

While this study provides a comprehensive synthesis of reported complication rates in thoracic MISS, several important limitations must be acknowledged. Most included studies were nonrandomized and retrospective in design, introducing potential selection bias and limiting the overall strength of the evidence. The small number of studies evaluating uniportal endoscopic spine surgery and biportal endoscopic spine surgery restricted meaningful comparison across surgical approaches, particularly given that the biportal literature was represented by a single small cohort, limiting generalizability for this technique. Interpretation was further constrained by heterogeneity in surgical indications, procedural complexity, and patient populations across studies. Several reports included multiple pathologies without stratifying complications by indication, precluding pathology-specific comparisons and limiting interpretability, consistent with limitations noted in prior cervical MISS literature [15].

Differences in surgeon expertise, particularly during the initial learning curve of thoracic MISS techniques, may have also influenced the observed complication rates. Revision status was not consistently reported across studies, precluding stratification of complications by primary versus revision surgery and representing a potential source of residual confounding. The lack of standardized definitions for complications and inconsistent reporting across studies further complicates direct comparisons. Additionally, retrospective complication reporting may underestimate true event rates, particularly for complications such as dural tears or minor cerebrospinal fluid leaks that may not be consistently detected or documented. Moreover, key clinical metrics such as intraoperative blood loss, postoperative pain scores, and length of stay were inconsistently reported, limiting the ability to compare perioperative recovery profiles. Publication bias is also a potential concern in highly specialized surgical fields, where favorable outcomes may be preferentially reported, although formal assessment is limited by the small number of available studies. Restriction to English-language publications may have introduced language bias and limited inclusion of relevant non-English studies.

Future research should prioritize multicenter, randomized controlled trials to validate these findings, evaluate long-term outcomes, and directly compare thoracic MISS techniques to both open and other minimally invasive approaches. Identifying predictors of specific complications and addressing the unique challenges of thoracic spine surgery will be critical to optimizing these advanced techniques and supporting broader adoption. Furthermore, this study focused exclusively on perioperative complications, leaving critical outcomes such as functional recovery, long-term quality of life, and adjacent segment disease unaddressed.

## 5. Conclusions

Thoracic MISS offers a minimally invasive alternative to traditional open procedures, with reported overall complication rates in the literature generally remaining low. Neural injuries and dural tears were the most frequently reported complications across thoracic MISS studies. Interpretation of differences across tubular retractor, UESS, and BESS approaches is limited by small study numbers. Future prospective studies are essential to confirm these findings, evaluate long-term outcomes, and facilitate broader adoption of thoracic MISS techniques.

## Figures and Tables

**Table 1:** Overview of Reported Complications in Studies of Thoracic Minimally Invasive Spine Surgery. Entries marked “0” denote that the complication was explicitly stated as absent, whereas “-“ indicates that the complication was not mentioned in the study.

Study	Approach	Primary Pathology Treated	Sample	Total Complication	Neural Injuries	Dural Tears	Symptomatic CSF Leak	Surgical Revisions
Ross et al., 2014 [4]	Tubular	Mixed (Degenerative/Tumor)	40	1	0	1	0	-
Xu et al., 2019 [18]	Tubular	Intradural Tumor	17	2	0	-	2	0
Zhao et al., 2017 [19]	Tubular	Extradural (OLF)	13	0	0	-	-	-
Afathi et al., 2015 [5]	Tubular	Intradural Tumor	13	0	-	-	-	-
Balasubramanian et al., 2021 [6]	Tubular	Intradural/Extradural Tumor	20	2	2	0	0	0
Wang et al., 2021 [7]	Tubular	Extradural Tumor	31	0	-	-	0	0
Kim et al., 2022 [20]	Uniportal Endoscopic	Extradural (OLF)	31	3	0	1	-	1
Ruetten et al., 2018 [8]	Uniportal Endoscopic	Extradural (Disk/Stenosis)	55	11	6	2	0	1
Deng et al., 2022 [9]	Biportal Endoscopic	Extradural (OLF)	14	6	2	0	1	-

**Table 2:** Quality assessment of included studies using the Newcastle-Ottawa Scale for nonrandomized designs ^1^.

Study	Design	Selection	Comparability	Exposure	Total Score
Ross et al., 2014 [4]	Retrospective Cohort	3	0	2	5
Xu et al., 2019 [18]	Retrospective Cohort	3	0	3	6
Zhao et al., 2017 [19]	Retrospective Cohort	3	0	3	6
Afathi et al., 2015 [5]	Retrospective Cohort	3	0	3	6
Balasubramanian et al., 2021 [6]	Retrospective Cohort	3	0	3	6
Wang et al., 2021 [7]	Retrospective Cohort	3	0	3	6
Kim et al., 2022 [20]	Retrospective Cohort	3	0	3	6
Ruetten et al., 2018 [8]	Retrospective Cohort	3	0	3	6
Deng et al., 2022 [9]	Retrospective Case–Control	3	1	3	7

^1^ A score 6 or greater is indicative of good study quality.

## Data Availability

Data sharing is not applicable to this article, as no new datasets were generated or analyzed during the current study.

## Supplementary Materials

The following supporting information can be downloaded at: https://www.mdpi.com/article/10.3390/jcm15010363/s1, Text S1: Search strategy. Table S1: PRISMA 2020 checklist.

## Abbreviations

The following abbreviations are used in this manuscript:

MISS	Minimally Invasive Spine Surgery
UESS	Uniportal Endoscopic Spine Surgery
BESS	Biportal Endoscopic Spine Surgery
CSF	Cerebrospinal Fluid
PRISMA	Preferred Reporting Items for Systematic Reviews and Meta-Analyses
NOS	Newcastle–Ottawa Scale
OLF	Ossification of the Ligamentum Flavum
