# Supplementary material for: Complications in Thoracic Minimally Invasive Spine Surgery (2013–2024): A Systematic Review
Source: J Clin Med. 2026 Jan 3;15(1):363. doi: 10.3390/jcm15010363 (PMC12786803; doi:10.3390/jcm15010363)
Supplement: Supplementary file 1 [file jcm-15-00363-s001.zip › jcm-3997662-supplementary/Supplementary File 1 Text S1.pdf]

Supplementary Materials

Text S1. Search strategy.
PubMed <January 2013 to March 2024>

1. "Minimally invasive"[All Fields] OR "MISS"[All Fields] OR "Minimally Invasive Surgical Procedures"[MeSH Terms] AND "tubular"[All Fields] OR "biportal"[All Fields] OR "uniportal"[All Fields]
2. "spin*"[All Fields] OR "Spine"[MeSH Terms] OR "spine surger*"[All Fields]
3. "Lumbosacral Region"[MeSH Terms] OR "Cervical"[All Fields] OR "thoracic"[All Fields] OR "lumbar"[All Fields]
4. "Postoperative Complications"[MeSH Terms] OR "Intraoperative Complications"[MeSH Terms] OR "complication*"[All Fields]

1 AND 2 AND 3 AND 4 - 185

OVID for Embase and Medline
Embase <1974 to 2024 March 29>
Ovid MEDLINE(R) ALL <1946 to March 29, 2024>

1. (((Minimally invasive or MISS).af. or exp Minimally Invasive Surgical Procedures/) and tubular.af.) or biportal.af. or uniportal.af. 6821
2. spin*.af. or exp Spine/ or spine surger*.af. 2077336
3. 1 and 2 2194
4. exp Lumbosacral Region/ or Cervical.af. or thoracic.af. or lumbar.af. 2107173
5. exp Postoperative Complications/ or exp Intraoperative Complications/ or complication*.af. 7489485
6. 4 and 5 511290
7. 3 and 6 1215
8. limit 7 to yr="2013 - 2024" 1051
9. remove duplicates from 8 685

Cochrane Library < January 2013 to March 2024>

1. (Complications):ti,ab,kw AND ("Minimally invasive Surgery"):ti,ab,kw AND (Spine):ti,ab,kw (Word variations have been searched) 50
